# Signaling pathways in rheumatoid arthritis: implications for targeted therapy

**DOI:** 10.1038/s41392-023-01331-9

**Published:** 2023-02-17

**Authors:** Qian Ding, Wei Hu, Ran Wang, Qinyan Yang, Menglin Zhu, Meng Li, Jianghong Cai, Peter Rose, Jianchun Mao, Yi Zhun Zhu

**Affiliations:** 1 State Key Laboratory of Quality Research in Chinese Medicine & School of Pharmacy, Macau University of Science and Technology, Macau SAR, 999078 China; 2 School of Basic Medicine, Guizhou University of Traditional Chinese Medicine, Guiyang, China; 3 Affiliated Hospital of Guangdong Medical University, Zhanjiang, China; 4 School of Biosciences, University of Nottingham, Loughborough, United Kingdom; 5 Department of Rheumatology, Longhua Hospital of Shanghai University of Traditional Chinese Medicine, Shanghai, 201203 China; 6 Shanghai Key Laboratory of Bioactive Small Molecules, Department of Pharmacology, School of Pharmacy, Fudan University, Shanghai, 201203 China

**Keywords:** Rheumatic diseases, Drug discovery

## Abstract

Rheumatoid arthritis (RA) is an incurable systemic autoimmune disease. Disease progression leads to joint deformity and associated loss of function, which significantly impacts the quality of life for sufferers and adds to losses in the labor force. In the past few decades, RA has attracted increased attention from researchers, the abnormal signaling pathways in RA are a very important research field in the diagnosis and treatment of RA, which provides important evidence for understanding this complex disease and developing novel RA-linked intervention targets. The current review intends to provide a comprehensive overview of RA, including a general introduction to the disease, historical events, epidemiology, risk factors, and pathological process, highlight the primary research progress of the disease and various signaling pathways and molecular mechanisms, including genetic factors, epigenetic factors, summarize the most recent developments in identifying novel signaling pathways in RA and new inhibitors for treating RA. therapeutic interventions including approved drugs, clinical drugs, pre-clinical drugs, and cutting-edge therapeutic technologies. These developments will hopefully drive progress in new strategically targeted therapies and hope to provide novel ideas for RA treatment options in the future.

## Introduction

**Fig. 1:**
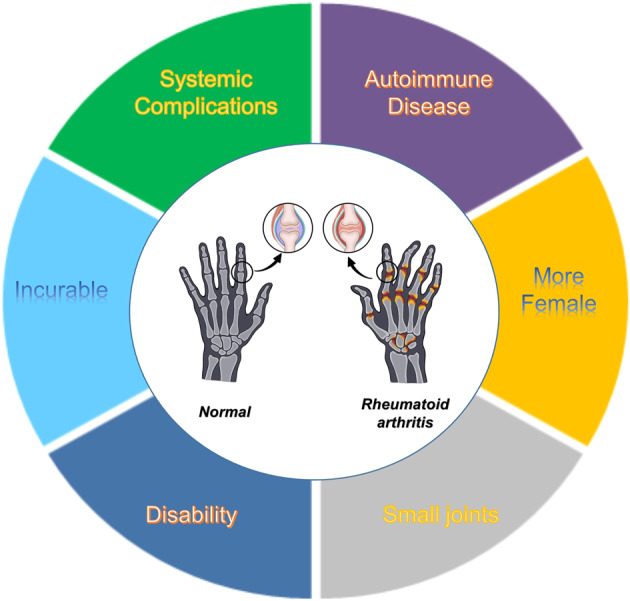
General features of rheumatoid arthritis. Rheumatoid arthritis is an incurable autoimmune disease that occurs most frequently in women, usually in the small joints, with systemic complications that ultimately lead to disability

Rheumatoid arthritis (RA) is a well-known systemic autoimmune disease. The general features of RA are demonstrated in Fig. 1. The original terminology for ‘rheumatoid arthritis’ is derived from the Greek word for inflamed and watery joints.^1,2^ The first person to describe and classify this debilitating disease was the French doctor Augustin Jacob Landré-Beauvais in 1880. Landré-Beauvais recorded the important manifestations of the disease with “asthenic gout,” indicating that the condition occurred well in women.^3^ Later, the British rheumatologist Dr. Alfred Baring Garrod coined the term “rheumatoid arthritis” in 1859.^4^ Critically, it is now known that global, the incidence of RA is ~1%,^5–10^ with prevalence increasing with age; the disease commonly comes up between the ages of 40 and 50 in individuals with the condition three to five times more in women than in men.^6,11–13^ Repeated and symmetrical multiple micro arthritis is the primary clinical manifestation of the disease, occurring in the hand, wrist, foot, knee, and other joints. In the early stages of the disease, redness, swelling, heat, pain, and joint dysfunction are common.^14^ The European League Against Rheumatism (EULAR) and the American College of Rheumatology (ACR) developed new classification criteria for RA: according to joint symptoms, serology indicators (RF or ACPA), duration of symptoms, acute phase reactants, each of these categories has scoring criteria.^15^ Methotrexate therapy was initiated by identifying disease characteristics, a consensus decision was made, and a scoring system was created to predict which patients would develop erosive and/or persistent disease.^15–17^ In the late stages of the condition, different degrees of rigidity and deformity of joints are seen and, finally, drive several degrees of bone corrosion and skeletal muscle atrophy, synovitis invasion of articular cartilage, sub-cartilage bone erosion, and damage to ligaments and tendons.^18^ The disease seriously affects the quality of daily life and suffers have high disability rates, and this can impact the loss of labor in the general population. RA also occurs in other tissues and organs, viz. extra-articular tissues and organs, including the eyes, nerves, skin, kidney, lungs, liver, heart, and bones.^19–22^

The cause of RA remains unknown, but it is generally considered related to environmental and genetic factors. The mechanism(s) of action include the joints attacking by body’s immune system by mistake, which causes joint capsule inflammation and thickening, and promotes damage to bones and cartilage at these sites. In the clinical, RA diagnosis is based on the patient’s physical manifestations and symptoms.^23–27^ X-rays and laboratory tests can assist in the diagnosis or exclusion of some similar disorders, viz. lupus erythematosus, psoriatic arthritis, and fibromyalgia.^28–33^ Since RA is incurable, it burdens individuals and society.^34–38^ The personal burden arises from musculoskeletal defects, accompanied by a decline in physical function and quality of life.^39,40^ In addition to direct medical costs, the socioeconomic burden results from RA patients having dysfunction and decreased working ability, and reduced social participation.^41^ A recent survey in China showed that the average annual direct cost per RA patient was $1917.21 ± $2559.06.^42^ The Burden of RA across Europe: a Socioeconomic Survey (BRASS) by using Work Productivity and Activity Impairment questionnaire (WPAI) scores indicated that RA people with severe (60%) or moderate pain (48%) experienced additional work obstacles compared with those with mild (34%) or no pain (19%), a statistically remarkable correlation was found between severity, pain, disability, and early retirement.^43^ Similar survey results in Latin America, China, and other Countries have also been reported.^44–46^

The pathogenesis of RA is tightly related to many characterized signaling pathways. Therefore, in recent years, research attention has focused on developing molecules that function as inhibitors of RA-linked signaling systems. This review will generally introduce the risk factor and pathogenesis of RA, mainly describing the signal transduction pathways that impact RA, drugs in clinical use, potential drugs in clinical/pre-clinical studies, and technologies for targeted therapy.

## Risk factors and symptoms

Over the past few decades, RA etiology has been numerously explored, and the available evidence indicating environmental and genetic factors are important in inducing RA. Indeed, the susceptibility genes HLA-DRB1, TNFRSF14, and PTPN22 are closely related to the occurrence of RA.^47–50^ HLA-DRB1 is the most widely explored gene and forms part of the HLA complex, the major histocompatibility complex (MHC) human version.^51–53^ The susceptibility and outcome of RA may be related to specific HLA-DR alleles; however, these alleles vary by ethnicity and geographic region.^54–56^ The HLA-DRB1 allele constitutes the strongest genetic association linked to RA, and the allele associated with the disease is a “shared epitope” with a conserved sequence of five amino acids.^47^ The shared epitope hypothesis indicates that some alleles with this conserved sequence are in connection with the pathogenesis of RA because they allow antigen-presenting cells to incorrectly present their antigens to T cells, which results in T-cell-mediated autoimmune responses that directly contribute to the RA pathogenesis.^57,58^ Environmental factors are also key points in causing RA, such as smoking, personal dietary pattern, and hygiene, which directly affects the post-transcriptional modification of certain genes or indirectly affects susceptibility genes via epigenetic mechanisms.^59–62^ The interaction of environmental factors, epigenetics, and susceptibility genes will drive changes in the relative levels and expression of coded proteins, which could promote autoimmune tolerance disorders.

**Fig. 2:**
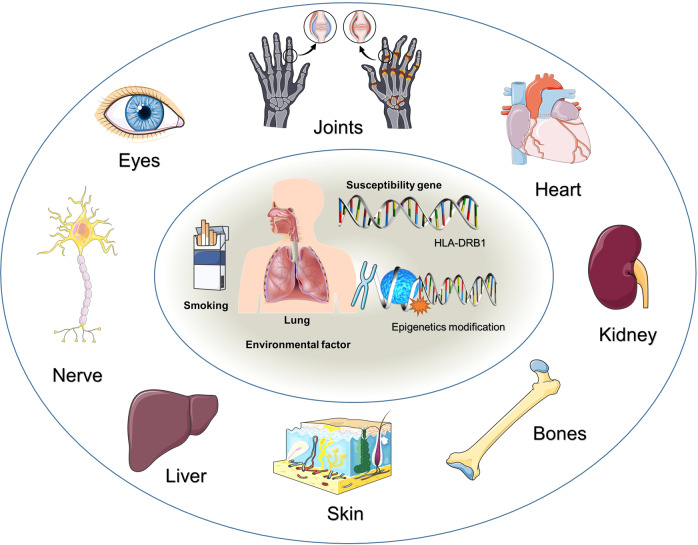
Risk factors and systemic complications of RA. Genetic and environmental factors are important in inducing RA. While RA mainly affects the joints, it can also influence other organ systems, including the eyes, nerves, skin, kidneys, lungs, liver, heart, and bones

While RA mainly affects the joints, it may also influence other organ systems,^63,64^ including the eyes, skin, lungs, liver, heart, and bones (Fig. 2). RA usually presents signs of inflammation, swelling, fever, pain, and stiffness in the affected joints. In general, these processes occur in the small joints of the feet and hands but may also occur in larger joints such as the shoulder and knees.^65–70^ These symptoms are more pronounced after long periods of inactivity, and a conspicuous feature of the disease is increased stiffness in the morning.^71–75^ Pain related to RA is caused at the inflammation site and is classified as nociceptive rather than neuropathic.^76,77^ As the pathological condition progresses, continued inflammation results in tendon binding and erosion and destruction of the articular surface; this can impair the range of motion and lead to deformity,^65,78–80^ and local osteoporosis often occurs around the inflamed joints of RA patients.^81,82^ Sustained production of inflammatory mediators creates a pro-inflammatory cycle, a situation common to many chronic diseases and this likely explains why RA patients are at greater risk of cardiovascular diseases.^83–86^ In addition, untreated chronic inflammation may lead to renal amyloidosis,^87,88^ rheumatoid nodules in the skin,^89–91^ and interstitial lung disease (ILD).^92–95^ Moreover, in the eye, episcleritis is common,^96–98^ liver problems like autoimmune hepatitis can also trigger problems,^99–101^ and peripheral neuropathy caused by wrist swelling and median nerve compression is a common problem in carpal tunnel syndrome. Rheumatic diseases of the spine can also contribute to myelopathy, atlantoaxial subluxation may occur due to erosion of the transverse ligament, and can progress to paralysis and even death.^102^

## The pathogenesis of RA

RA is initially a state of continuous cellular activation that results in autoimmunity in joints or other organs.^103,104^ The clinical manifestations of the disease occur predominantly following synovial inflammation and joint injury. Fibroblast-like synoviocytes (FLS) play a crucial role in these pathological courses.^105–107^ Three stages of RA progression are reported and include a non-specific inflammatory stage, amplified by T-cell activation in the synovium, the chronic inflammatory stage, and a tissue damage stage mediated by cytokines like IL-1, IL-6, and TNF-α, respectively.^108–111^

### Autoimmune response and inflammation

The production of autoantibodies has been linked to severe symptoms like joint injury and increased mortality.^112–116^ This is likely because of the generation of immune complexes by autoantibodies against citrullinated peptides (ACPAs) with citrulline-containing antigens. These complexes subsequently bind to rheumatoid factors (RF), leading to complement activation.^117–122^ In recent times, the capacity to detect autoimmune responses to citrullinated self-proteins has been a major advance.^123^ In RA patients, the degree of association between ACPA-positive and ACPA-negative and shared epitopes are different. The non-HLA genes correlated to RA susceptibility between the two genomes are only partially the same. Therefore, some researchers believe ACPA-positive and ACPA-negative RA may be two genetically distinct disease types of RA.^124^ Some studies have shown that when certain factors in the environment change, arginine is converted into citrulline under the catalysis of peptidylarginine deiminases (PADs), and citrullinated proteins can, through antigens presenting cells (APCs) present to T cells by certain MHC, produce ACPAs and simultaneously elicit autoimmune responses to citrullinated self-antigens in RA patients.^125,126^ Peptidyl arginine deiminase type4 (PADI4) is also identified as the non-MHC genetic risk factor of RA. Meanwhile, the PADI4 risk allele was associated with bone damage regardless of ACPA positivity in Asian RA patients.^127^ ACPA binds citrullinated residues on many of the body’s own proteins, including histones, vimentin, fibronectin, fibrinogen, type II collagen, and alpha-enolase, the activated immune responses tissue is uncertain.^128^ Circulated ACPAs could be detected up to 10 years before diagnosis known as pre-RA.^129–133^ As time progresses, the epitope diversity and concentration of ACPAs increase, and so do the concentrations of serum cytokine. With effective treatment, ACPA and RF concentrations decrease; however, patients rarely turn into ACPA negative. In contrast, RF drops are more profound and more frequent, and the patients may seroconvert to RF negativity.^134^ Anti-carbamylated protein (CarP) and acetylated protein autoantibodies also have been identified in RA patients^135,136^; moreover, other-directed against additional post-translational protein modifications autoantibodies may emerge.

**Fig. 3:**
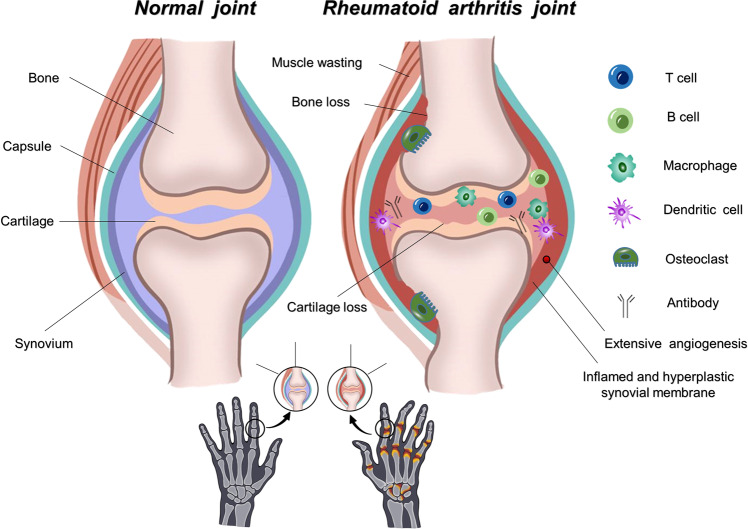
Normal and rheumatoid arthritis joints. Joint swelling in RA reflects synovial inflammation due to immune activation. The cellular composition of RA synovitis is characterized by the accumulation of innate and adaptive immune cells (e.g., T cells, dendritic cells, B cells, macrophages, and osteoclasts). Pro-inflammatory and bone-destructive factors of the immune response led to the loss of bone or cartilage with synovial thickening, angiogenesis, and muscle wasting

Joint swelling in RA is the result of synovial inflammation caused by immune activation. The swelling is characterized by the entry of leukocytes into the synovial compartment. The cellular composition of RA synovitis is manifested by the accumulation of innate immune cells (e.g., dendritic cells, monocytes, mast cells, and innate lymphoid cells) and adaptive immune cells (e.g., T-helper-1 and T-helper-17 cells, B cells, plasmablasts, and plasma cells). Innate immunity can be initiated by provoking dendritic cells (DCS) in certain environmental or genetic factors. DCs then recruit and activate T cells, which stimulate B cells, macrophages, synoviocytes, chondrocytes, and osteoclasts, and secrete pro-inflammatory and bone-destroying cytokines i.e., IL-1β, IL-6, TNF -α, and matrix metalloproteinases (MMPs).^137–139^ Therefore, in the adjacent bone marrow and synovium, the integration of innate and adaptive immune pathways promotes tissue injury and remodeling.^140^ This cascade drives chronic inflammation in RA and promotes circulating leukocytes to migrate into the inflamed joint; this process needs angiogenesis to supply nutrients and oxygen to the hypertrophic joint. Proangiogenic factors trigger angiogenesis.^141–143^ Fibroblast-like synoviocytes (FLS) in the synovium intima form a unique invasive phenotype that promotes extracellular matrix invasion and further exacerbates joint injury.^144–146^ (Fig. 3).

### FLS and immune cells in RA

Currently, studies on RA have analyzed the character of immune cells in the occurrence and course of the disease. More recently, attention has also focused on the local interstitial cells and the role these cell types play in the pathogenesis of RA. Stromal cells constitute the structural framework of organs and tissues.^147^ Stromal cells are thought to have immune functions, can recognize pathogens, and trigger immune responses. Fibroblasts of the intestine, skin, gums, and synovium are typical stromal cells. They have been proven to express innate immune receptors, especially Toll-like receptors (TLR).^148–152^ These stromal cells present and express antigens through histocompatibility complex (MHC) II receptors and secrete cytokines and chemokines. Therefore, these stromal cells are components of the innate immune system.

In non-pathological synovial tissue, the normal physiological function of FLS is to build the lining layer of the synovium, secrete synovial fluid, lubricate proteins in the joint, and provide plasma protein for the adjacent cartilage and joint cavity.^153^ In addition, FLS participates in the continuous remodeling of the synovium by producing matrix components, like collagen, thereby maintaining synovium homeostasis. Under the conditions of RA inflammation, FLS undergoes a profound change from harmless mesenchymal cells to destructive and aggressive tumor-like cells. These transformed RA FLS play a leading role in the production and progression of RA and show a special phenotype characterized by reduced sensitivity to apoptosis, overexpression of adhesion molecules, and abnormal production of cytokines, chemokines, and matrix metalloproteinases (MMPs).^149,154,155^

**Fig. 4:**
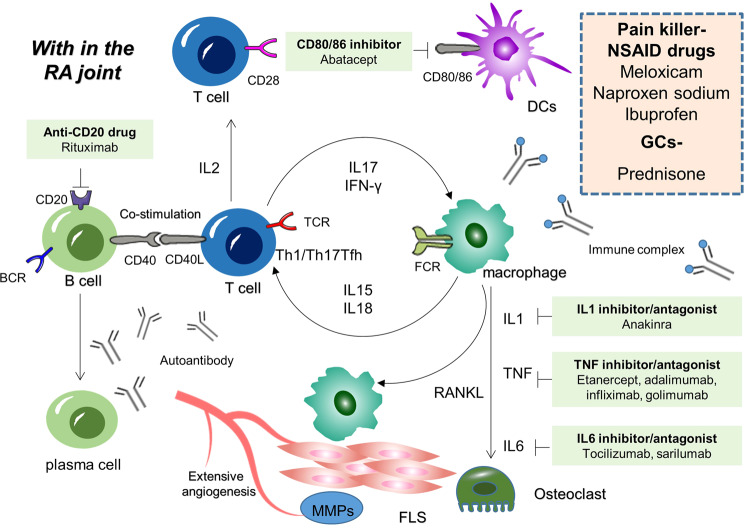
Cytokine signaling and anti-rheumatic drugs in RA. In the presence of certain environmental or genetic factors, a stepwise progression from activation of innate immunity can be achieved by stimulating DCs, then recruiting and activating T cells, which in turn stimulate B cells, macrophages, synoviocytes, chondrocytes, and osteoclasts to secrete pro-inflammatory and bone-destroying cytokines (i.e., IL-1β, IL-6, TNF-α, and matrix metalloproteinases (MMPs), resulting in bone and cartilage damage accompanied by synovial membranes thickening and angiogenesis, in the synovium and adjacent bone marrow, and the integration of adaptive and innate immune pathways to promote tissue remodeling and damage drives the chronic phase of RA. Clinically, drugs that target inflammatory cytokine signaling are commonly applied

A complex network of cytokine and chemokine regulates the synovial compartment inflammatory environment; several clinical interventions (Fig. 4) suggest that among these components, granulocyte-monocyte colony-stimulating factor, interleukin-6 (IL-6), and tumor necrosis factor (TNF) are essential to the process.^156,157^ Cytokines and chemokines promote inflammation by activating endothelial cells, attracting immune cells accumulation in the synovial compartment, activating fibroblasts, and accumulating activated T cells and B cells. Activated B cells with the assistance of antigen-presenting cells and Th cells, then differentiate into plasma cells to synthesize and secrete various immunoglobulins, helper T cells (Th) differentiate into, Th1, Th2, Th17, and Treg cells on the basis of the cytokine microenvironment. Th1 cytokine secretion was observed to contribute to the increase of Th17 infiltration and IL-17 production in synovial tissues during RA.^158^ Follicular helper T (Tfh) cells, a subset of CD4+ T cells, promote germinal center (GC) responses by providing the signals needed for high-affinity antibody generation and production of long-life antigen-secreting plasma cells.^159^ In various systemic autoimmune diseases, uncontrolled expansion of Tfh cells has been observed, and in particular, the frequency of circulating Tfh-like cells, their subtypes, and synovially infiltrating T helper cells correlates with the disease process in patients with RA^160^; Osteoclast generation is triggered by monocytes and macrophages via receptor activator of nuclear factor κB ligand (RANKL), and fibroblasts following direct interaction with the RANK receptor on dendritic cells, macrophages, and pre-osteoclasts.^161–163^ Bony erosions occur at the so-called bare area of the junction between cartilage, periosteal synovial membrane, and bone.^164^ Cytokines bind to homologous receptors to trigger plenty of intracellular signal transduction events, causing the activation of genes coding for systems that can aggravate inflammation and cellular and tissue damage.^165^

## Signaling pathways in the pathogenesis of RA

**Fig. 5:**
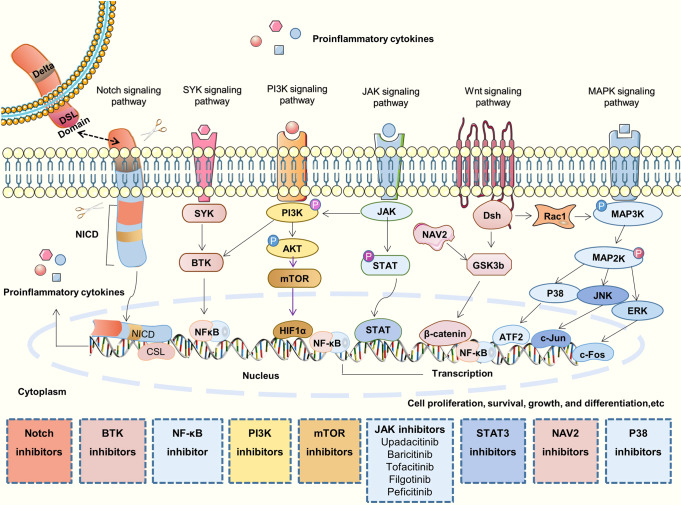
Main signaling pathways and their inhibitors related to RA. The JAK signaling pathway, Notch signaling pathway, MAPK signaling pathway, Wnt signaling pathway, PI3K signaling pathway, and SYK signaling pathway are the main signaling pathways involved in the process of RA. Related signalings are often the potential targets for drug discovery

Multiple signal transduction pathways are involved in the disease progression of rheumatoid arthritis, the major signaling pathways are shown in Fig. 5, and the abnormal signals are often targets for drug discovery.

### The JAK-STAT signaling pathway

The JAK (Janus-activated Kinase)- STAT (Signal Transduction and Activator of Transcription) is one of the most crucial signaling pathways for cytokine signaling, with well-known regarding how TNF-α rapidly induces the target genes expression, for example, the interferon family, gp130 family, common-γ chain family, receptor tyrosine kinases, and some G protein-coupled receptors can induce transduction through the JAK-STAT pathway.^166^ This signaling pathway is believed to play a crucial character in cell differentiation, proliferation, apoptosis, and immune function and is especially important in regulating inflammation and immune function. Numerous recent studies found that the JAK-STAT signaling pathway is abnormally activated during RA.^167–169^ JAK family has four members: JAK1, JAK2, JAK3, and TyK2 (tyrosine Kinase 2). The four members have different molecular weights and are highly conserved in the evolutionary process. Although JAK3 is only expressed in blood, vascular smooth muscle, and endothelial cells, JAK1, JAK2, and TyK2 are all widely expressed in multi-tissue and multi-system.^170,171^ Studies have shown that JAK plays an important role in RA.^172–174^ STAT is a family of cytoplasmic proteins with both transcriptional activation and signal transduction functions. The STAT family including STAT1-4, STAT5A, STAT5B, and STAT6. STAT contains six highly conserved functional domains, the N-terminal conserved domain, the helix domain, the DNA-binding domain, the ligation domain, the SH2 domain, and the C-terminal transcriptional activation domain.^175,176^ Among these regions, the most conserved and functionally important domain is the SH2 domain. The SH2 domain allows for the specific recognition and docking of phosphorylated tyrosines on the cytokine receptors, JAK, and other STAT molecules.^177^ The N-terminal is important in controlling STAT interaction with other transcription factors while the DNA-binding region determines where STAT interacts with DNA.^178^

In recent years, the occurrence and progression of RA have been considered highly correlated to the abnormal activation of the JAK-STAT pathway. This pathway is involved in many pathological conditions and seems important in abnormal hyperplasia of RA FLS, synovial inflammation, and bone destruction.^179–181^ Among these components, synovitis is the pathological basis of RA. Persistent synovitis leads to abnormal hyperplasia of synovitis, which leads to the destruction of bone and cartilage. Many inflammatory reactions have been observed in RA synoviums, such as the activation of adhesion molecule genes and cytokines, which are closely correlated to transcription factors in specific signaling pathways.^182,183^

JAK1 is involved in signal transduction associated with various cytokines, like IFN-γ and IL-6, that bind and form receptor complexes that activate JAK1 kinase and take part in the pathogenesis of RA, vitiligo, and psoriasis.^184,185^ JAK3 is participated in the signaling pathways linked to IL-2, IL-4, IL-7, IL-9, IL-15, and IL-21, and plays an important role in the differentiation, proliferation, and growth of T cells.^168^ Tyk2 could be activated by IFNs, IL-6, IL-10, IL-12, IL-23, and selective inhibition of Tyk2 plays a role in the RA treatment.^186^ JAK2 induces downstream activation of STAT3 and STAT5. It is responsible for signaling through multiple receptors, including receptors that play a function in inflammatory and autoimmune responses, such as IL-6R, IL-12Rβ, and IFN-γ R2. JAK2 is known to be related to a variety of diseases, including blood diseases, diabetes, cancer, and autoimmune diseases. Compared with normal healthy people, the expression of JAK2 in synovial tissue of RA patients is significantly increased.^187^ Similar expression patterns are also reported in collagen-induced arthritis rats, adjuvant arthritis rats, and other animal models. Indeed, Kristine S et al. used CEP-33779 (highly selective JAK2 inhibitor) to intervene in collagen antibody-induced and collagen-induced mouse arthritis models. The levels of cytokines (IFN-γ, IL-12, and TNF-α) and serum IL-2, IL-12, and p-Stat 3 in the synovial fluid of the model mice were significantly decreased following treatment. Indeed, CEP-33779 significantly reduced several histological parameters that demonstrated improvement in arthritis, including matrix erosion, subchondral osteolysis, osteogenesis, synovial hyperplasia, vasculitis, and synovial inflammation.^188^ These results indicated that JAK2 took part in the pathogenesis of RA, and inhibition of JAK2 could treat RA by inhibiting the generation of cytokines and the T and B-lymphocytes activation. Additionally, receptors phosphorylated by JAKs can also recruit PI3K, thereby activating the PI3K-AKT pathway.^182,189^

STAT1 is mainly activated by cytokines such as IL-6, IL-10, and IFN-γ and participates in body activities through IFN-γ-mediated signaling pathways. Currently, STAT1 has been proven to play both protective and pathogenic roles in RA synovitis. Still, its expression generally rises in inflammatory arthritis, indicating that the anti-inflammatory and pro-apoptotic effects of STAT1 are insufficient to counteract its pro-inflammatory effects. IFN-λ and IFN-α/β only activate STAT2. It has been shown that STAT2 is involved in RA-associated inflammation through the combination of STAT1 and interferon regulatory factor 9 (IRF-9) to form a heterodimer transcription complex of interferon-stimulated gene factor (ISGF3).^190^ STAT4 regulates the balance of IL-12 and IL-23 and participates in RA inflammation through the differentiation of CD4+ T cells into Th17 and Th1 cells. Multiple meta-analyses results^191,192^ have shown that a single nucleotide polymorphism at rs7574865 of the STAT4 gene potentially correlated with RA susceptibility however, more research is needed to confirm this observation. Other components like STAT5, a Treg cells transcription factor, which, together with Foxp3, is responsible for the differentiation of Treg cells. Various studies have found that the effect of STAT5 may be opposite to that of STAT3 since the inhibition of STAT3 can increase the activity of STAT5 and Foxp3. Thus, it can promote the differentiation of Treg cells and control RA arthritis.^193^

Similarly, STAT6 is activated by IL-4 while IL-13 is activated by IFN-α in B-lymphocytes. In a proteoglycan-induced mouse arthritis model, IL-4 and STAT6 deficiency can significantly increase the severity of arthritis.^194^ STAT3 is the primary downstream regulator of the gp130 receptor and can be activated by IL-6, IL-10, IFN-α/β, and other cytokines. It can promote chronic arthritis, regulate the abnormal growth and survival characteristics of RA synovial cells, and further aggravate the clinical symptoms of RA. Wang et al.^195^ and colleagues were the first to observe that STAT3 showed DNA-binding activity in synovial mononuclear cells from patients with inflammatory arthritis. Moreover, Lee et al.^196^ found that STAT3 can inhibit FLS apoptosis, increase the activity of T cells and promote the production of antibodies, indicating that STAT3 is involved in multiple links of RA pathogenesis. Oike et al.^197^ found that in collagen-induced arthritis model mice, p-STAT3 was highly expressed in synovium and cartilage. In addition, the inflammatory cytokines IL-17 and IL-6 in serum were significantly reduced after STAT3 inhibitor treatment. Ji Hyeonet al.^193^ found that STAT3 was strongly expressed in both RA CD4+ T and synovial cells. The activation of STAT3 made synovial cells have tumor-like characteristics and made synovial cells proliferate fast, survive long, and infiltrate surrounding joint tissues. Notably, STAT3 plays an important role in determining RA helper cell differentiation. Indeed, transfection of STAT3 siRNA inhibited CD4+ T-cell differentiation into Th17 cells and increased the proportion of Treg cells.^198^ All these results show that STAT3 is closely related to articular inflammation and lymphocyte differentiation, and STAT3 might be a new target for the treatment of RA.^197^

### The MAPK signaling pathway and RA

The MAPK (Mitogen-Activated Protein Kinase) signaling pathway contributes to the regulation of various cellular activities, including gene expression, metabolism, migration, survival, cell cycle progression, apoptosis, and differentiation, which plays a key role in the pathological process of RA.^199^ Its overactivation is closely correlated to the articular cartilage destruction and inflammatory hyperplasia of the synovial tissues. MAPK regulates the expression of multiple genes and has been considered a potential target for treating RA or other immune-mediated chronic inflammatory diseases.^200^ It can transduce extracellular signals such as growth factors, neurotransmitters, hormones, stress conditions, viruses, and inflammatory factors into the cells^201,202^ and play a key role in the transduction of extracellular stimulation to drive intracellular responses.^203^ P38 MAPK, extracellular signal-regulated kinase (ERK), and c-Jun N-terminal kinase (JNK) are the three main subfamilies of the MAPK pathway.^204^

The ERK1/2 activates stimuli in response to ischemia, oxidative stress, and neurotransmitters. ERK1 and ERK2 are key to the regulation of cell differentiation, proliferation, and survival.^205^ The main effect of JNK MAPKs in RA is cartilage destruction mediated by matrix metalloproteinase (MMP).^206^ Similarly, P38 is the most important member of the MAPK family linked to the inflammatory response in rheumatoid arthritis. After inflammatory stimulation, p38 is activated and induced in endogenous immune cells such as neutrophils and monocytes. P38 then undergoes nuclear translocation, where it phosphorylates and activates many protein kinases and transcription factors that play key roles in the regulation of humoral and cellular autoimmune responses. In the synovial tissue of RA, p38 is activated and highly expressed by MKK3 and MKK6,^207^ and commonly used p38 MAPK inhibitors reduce the generation of pro-inflammatory cytokines in neutrophils, macrophages/monocytes, and T lymphocytes.^203^ P38 MAPK activates and moves into the nucleus, phosphorylating transcription factors such as ATF2, MEF2C,^208,209^ and these initiate cascades that induce a large increase in inflammatory chemokines like IL-8 and monocyte chemoattractant protein-1 (MCP-1), resulting in synovial thickening. p38 MAPK can also inhibit cell apoptosis. Yu et al.^210^ found that Integrin activation-induced phosphorylation of p38 MAPK inhibited Fas protein-mediated-cell apoptosis, resulting in a large number of T cells infiltration in synovial tissue, aggravating RA patients’ disease process. Many T cells infiltrate the synovial tissue, most of which are helper T (Th) cells. Studies have shown that the imbalance between Th1/Th2 cells is a crucial pathogenic factor in RA, and the imbalance of Th1/Th2 cells leads to IL-2 and IL-4, and other cytokines being abnormally secreted. Pujari et al. found that Th1/Th2 cytokine secretion is achieved through the P38 MAPK pathway, and inhibition of p38 activity can change the balance of natural CD4+ T cells preventing the differentiation of Th1/Th2 cell types viz. to inhibit their differentiation to Th1 cell variants. Th17 is a newly discovered helper T-cell subset and is featured by the secretion and production of the inflammatory factor IL-17. In the occurrence and development of RA, the role of Th17/IL-17 is also controversial.^211^ Hot et al.^212^ found that IL-17 isoform IL-17A can induce three signaling pathways of MAPK family ERK, p38, and JNK and downregulate transcription factors p65 NF-κB and AP-1. These indicate that MAPK and T-cell-mediated RA have a very complex relationship.^213^ Therefore, P38 is considered a candidate target for treating rheumatoid arthritis.^214^

### The PI3K-AKT signaling pathway in RA

The PI3K (phosphatidylinositol 3 kinase)-AKT (also known as PKB) pathway is an intracellular pathway that regulates proliferation, metabolism, angiogenesis, and cell survival in response to extracellular signals. The key involved genes are PI3K and Protein kinase B (PKB).^215–217^ PI3K can phosphorylate PIP2 to PIP3 by adding a phosphate group, and phosphatases, such as PTEN, can dephosphorylate PIP3 back to PIP2.^218^ This cycle thereby terminates PI3K signaling. The downstream effects of PI3K are mainly reflected in the regulation of PIP3. In the PI3K-AKT pathway, the phosphate group at position 3 of PIP3 can simultaneously recruit PDK1 and AKT proteins to the plasma membrane, causing PDK1 to phosphorylate threonine at position 308 (T308) of AKT protein. This contributes to the activation of AKT, which further activates the downstream regulatory pathways.^215,219^

It has been proven that the PI3K/AKT signaling pathway is correlated with the occurrence and development of RA. It can participate in the unusual proliferation of FLS cells and synovial inflammation by stimulating the expression of inflammatory molecules like IL-1β, IL-6, IL-17, IL-21, IL-22, and TNF-α, which constitute the most important pathogenesis of RA pathological changes.^220–224^ IL-17, TNF-α, and other cytokines are involved in the generation of osteoclasts, which destroy articular cartilage and bone, resulting in joint stiffness and deformity.^225^ Abnormal PI3K/AKT pathway activation will also stimulate the expression of VEGF and HIF-1α to promote angiogenesis, which not only isolates bones from getting nutrients through the synovium but also gets involved in the release of diverse inflammatory mediators, aggravating the condition of RA.^226–228^ There is evidence indicating that the PI3K/AKT/mTOR pathway participates in the process of RA, the mammalian target of rapamycin (mTOR) inhibits autophagy in FLS, promotes continuous abnormal proliferation of synovial cells, and is also critical for the survival and differentiation of osteoclasts, aggravating RA and mTOR might be a target for RA or other autoimmune diseases.^227,229–231^

### SYK signaling pathway in RA

SYK (spleen tyrosine kinase) is a central molecule of B-cell receptor signaling. The level of phosphorylated SYK in peripheral blood B cells of RA patients is dramatically increased. Among these patients, also show strong positive autoantibodies against citrullinated peptides.^232^ B cells and autoantibodies are produced in most patients of RA and play a crucial role in the pathogenesis of RA.

BTK (Bruton’s tyrosine kinase) belongs to the Tec family of non-receptor tyrosine kinases, which is expressed in all hematopoietic cells, such as B cells and myeloid cells, except T cells and natural killer cells. BTK is a key molecule linking B-cell receptor (BCR) signaling, chemokine receptor signaling, and Toll-like receptor (TLR) signaling, and is involved in regulating B cells.^233^

In antigen-dependent BCR signaling, BTK can be activated by SYK or PI3K and participate in regulating B-cell survival and proliferation.^234^ In antigen-independent TLR signaling, most TLRs recruit MYD88 in response to the TLR ligand.^235^ In chemokine receptor signaling, CXCL12, which is overexpressed in the germinal centers and bone marrow, directly interacts with CXCR4-linked heterotrimeric G protein subunits through BTK, binds to CXCR4, and induces BTK activation.^236^ In addition, BTK can directly interact with five distinct molecules to promote antibody secretion, class switch recombination, cell proliferation, and generation of pro-inflammatory cytokines, regulating B-cell migration, adhesion, and tumor microenvironment forces.^233^ As previously described, RA is a systemic autoimmune disease involving dysregulation of T and B lymphocyte proliferation, and dysregulation of B cells via BCR signaling drives the generation of autoantibodies and inflammatory cytokines, thus promoting the progression of RA.^237^ Elevated levels of phosphorylated BTK have been found in peripheral B cells of RA patients. Meanwhile, in RA patients with rheumatoid factor (RF) positive, the level of phosphorylated BTK was correlated with RF titer.^237,238^ BTK mediates bone resorption by RANK and regulates osteoclast proliferation and differentiation, which is the main factor in the pathophysiological level of BTK phosphorylation by peripheral B cells in RA patients.^237,239^ Therefore, BTK is one of the most attractive targets for treating autoimmune diseases including RA.^240–243^

### Wnt signaling pathway in RA

The Wnt (Wingless/Integrated) signaling pathway is a complicated protein network that normally functions in cancer and embryonic development.^244^ The Wnt/β-catenin signaling pathway is activated and takes part in a variety of pathological symptoms such as maintenance, differentiation, proliferation, and self-renewal in RA.^245^ Wnt also plays a key role in synovial inflammation and in the regulation of bone metabolism in RA.^246^ Wnt family secreted proteins, Frizzled family transmembrane receptor protein Dishevelled (Dsh), glycogen synthesis kinase 3 (GSK3), β-catenin, APC, Axin, and TCF/LEF family transcriptional regulators constitute the classical wnt pathway^247^; In the non-classical Wnt pathway such as Wnt-Frizzled/PCP signal conduction, Dsh signals through the Rac1 axis and Daam1-RhoA axis.^248^

NAV2 belongs to the neuro-guiding protein family, and the proteins encoded by it contain multiple functional domains, such as the CH domain, CC domain, CSID domain, and AAA domain.^249^ These functional domains involve a series of cellular processes, including signal transduction, gene expression regulation, protein degradation, membrane fusion, microtubule and filament dynamics, and cell migration.^250–253^ If the above cellular processes are abnormal, they may affect the normal cell function of biological individuals and lead to diseases. Previous studies have proven that NAV2 plays a key role in the development of the mammalian nervous system, resulting in abnormal nerve fiber density and in causing developmental defects of nerves in early embryos following NAV2 deletion.^254^ NAV2 is also an indispensable protein molecule in the outward growth of human neuroblastoma cells induced by all-trans retinoic acid.^255^

Our group demonstrates for the first time that NAV2 promotes the fibrocyte-like synoviocytes inflammatory response by activating Wnt/β-catenin signaling^256^ and the SSH1L/Cofilin-1 signaling pathway in rheumatoid arthritis. We also hypothesized that NAV2 might affect inflammation during RA disease progression and the cell-cell interaction in sensitizing joint-innervating neurons that contribute to arthritis pain.^257^ Although our studies first indicate that inhibition of NAV2 expression prevents RA progression and reverses inflammation-related phenotypes, we proposed that NAV2 is a novel promising intervention target for RA treatment.

### Notch signaling pathway in RA

Notch genes encode a class of cell-surface receptors which is highly conserved and regulate the development of cells in various organisms, from sea urchins to humans. Notch signaling affects numerous processes of normal cell morphogenesis, including cell proliferation, the differentiation of pluripotent progenitors, apoptosis, and the formation of cell boundaries.^258^ The Notch signaling pathway comprises Notch receptors, Notch ligands (DSL proteins), CSL (CBF-1, Suppressor of hairless, Lag), DNA-binding proteins, other effectors, and Notch regulatory molecules. Mammals have 4 Notch receptors (Notch-1-4) and 5 Notch ligands (Delta-like 1, 3, 4, Jagged1, and Jagged2). The Notch signal is generated by interacting with the Notch ligand of the adjacent cell and the receptor. The Notch protein undergoes cleavages and released the Notch intracellular domain (NICD) into the cytoplasm, followed enters into the nucleus to combine with the transcription factor CSL to form NICD/ CSL transcriptional activation complex activates the target genes of the basic-helix-loop-helix (bHLH) transcriptional repressor family such as HES, HEY, and HERP, and plays a biological role.^259^ Notch signaling expression and activation stimulate synoviocytes, macrophages, and fibroblast-like synoviocytes to secrete pro-inflammatory cytokines that exacerbate RA.^260–263^ Th17 cell differentiation is impaired when blocked Notch signaling.^264–266^ Notch-1 directly binds to the IL-17 and ROR-γT promoters to regulate Th17 differentiation.^267^ Notch-3 plays a key role in the antigen-specific T-cell differentiation, and Notch-3 blockade inhibits Th17 and Th1 cell activation in CIA mice.^268^ Notch-3 was also found to be remarkably upregulated in synovial fibroblasts, and in mice model, blocking Notch-3 signaling reduces inflammation and prevents joint injury.^269^ Targeting Notch was found to minimize associated tissue damage while reducing inflammation.^262,270^

### NF-κB and other transcription factors in RA

A variety of transcription factors, like NF-κB, Nrf2, HIF, and AP-1, are closely related to the pathogenesis of RA.^271^ The expression of NF-κB in the synovium of RA patients was significantly increased. Activated NF-κB induces the generation of several pro-inflammatory cytokines, such as IL-1β, IL-6, and TNF-α, thus accelerating the development of RA. Upregulation of pro-inflammatory cytokines could also modulate NF-κb activation through positive feedback, thereby, a vicious loop is formed, which intensifies RA development.^272,273^ At the same time, excessive NF-κB activation also induces apoptosis of abnormal FLS cells in RA.^274^ In the RA synovium inflammatory microenvironment, aberrant apoptosis of FLS is the major factor associated with RA synovium hyperplasia. In FLS, abnormal cell apoptosis further accumulates in joint tissues and debris adheres to cartilage and bone, exacerbating the articular cartilage and bone destruction.^275^ The expression of NF-κB-dependent genes further activates NF-κB, translocations NF-κB to the nucleus, and induces the target genes expression. HIF is critical for activating inflammatory cells and angiogenesis in RA.^276^ Ap-1 regulates MMP, cytokine production, and synovial hyperplasia, which is also an essential process in RA.^277,278^ The transcription factor Fra-1 enhances macrophage-mediated arthritic inflammation by targeting arginase 1.^279^ Nrf2 is related to chondrogenesis, prostaglandin secretion, osteoblast formation, and ROS production in RA.^280^ Thus, targeting transcription factor signaling represents a useful treatment strategy for RA. It has been reported that inhibition of NF-κB can inhibit inflammation, angiogenesis, pannus formation, leukocyte maturation, and activation, and osteoclast differentiation, targeting HIF-1α can induce dysregulation of MMP production, inflammatory cell recruitment, and angiogenesis, inhibition of AP-1 can inhibit the production of MMP-1, MMP-3, MMP-9, MMP-13, and IL-1β.^271,281^ New agents that regulate transcription factor pathways will be potential candidates for treating RA.

The transcription factor GATA4 is an important regulator of the expression of genes specific to cardiac differentiation. Our research group found increased levels of GATA4 in the synovium of patients with RA. This study is the first time to demonstrate that GATA4 plays a key role in regulating VEGF from RA FLS to induce cell migration, promote cell proliferation, and the formation of angiogenic tubes.^282^ In addition, this study provided evidence that GATA4 has a previously unknown function as a modulator of RA angiogenesis, and data validate GATA4 as the therapeutic target in RA mice. E2F1, the first transcription factor discovered in the E2F family, mainly exists in dimer binding with Dimerization proteins (DP), which could bind to the promoter region of target genes and regulate the transcription of target genes.^283^ We found that the transcription factor E2F1 can bind to the Neuron Navigator 2 (NAV2) promoter region, activate NAV2 transcription and expression, and regulate RA through the Wnt/β-catenin signaling pathway.^256,284^ At the same time, the STAT3-NAV2 axis was found to be a novel therapeutic target for rheumatoid arthritis by activating the SSH1L/ cofilin-1 signaling pathway,^285^ which may provide therapeutic avenues for reducing pain in RA patients.

## Epigenetics regulation in RA signaling pathway

**Fig. 6:**
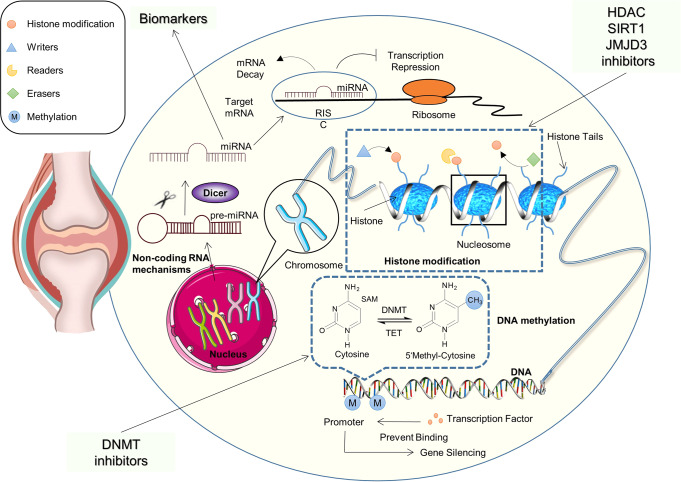
Epigenetic modifications and rheumatoid arthritis. DNA methylation, histone modifications, and-coding RNA mechanisms are often involved in the development of RA

Epigenetics are heritable changes of gene expression without altering the DNA sequence; epigenetics determines which genes are turned on or off. The main mechanisms linked to this process include histone modification, DNA methylation, and non-coding RNA mechanisms.^286^ These modifications define specific gene expression patterns (Fig. 6). Genetic and environmental factors interact to determine gene expression, especially, cigarette smoking,^287,288^ a lifestyle that is closely related to the pathogenesis of RA.^289–291^ Fortunately, these epigenetic modifications could be reversed, and the corresponding enzymes which control histone modification or DNA methylation have now been proposed as drug targets for RA.^292–294^

### Histone modifications and RA

Histones are proteins that help DNA package to form nucleosomes, and these structures further assemble into chromosomes in the nucleus of cells. Histone modifications at the N-terminal tail include ubiquitination, acetylation, methylation, phosphorylation, deamidation, and ADP ribosylation.^295^ The key modification sites are lysine and arginine. Histone modifications could inhibit or activate gene expression. Currently, many studies have applied histone modification to multiple diseases, especially in the field of cancer research, which offers new ideas for treating these diseases. Several studies have compared the differences in histone lysine methylation patterns between Osteoarthrosis synovial fibroblasts (OASFs) and Rheumatoid arthritis synovial fibroblasts (RASFs). These studies have detected dozens of histone lysine methyltransferases (HKMTs) and histone lysine demethylases (HKDMs). The results indicated that the expression of HKMTs and HKDMs in OASF and RASF were different at the mRNA level, suggesting that histone lysine methylation (HKM) could influence RASF gene expression.^296^ NAD-dependent deacetylase sirtuin-1 (SIRT1) is the most frequently explored member of the Nuclear-localized type III histone deacetylases (Sirtuin) family. SIRT1 is participated in several stages of rheumatoid arthritis, in which overexpression leads to-inflammatory cytokine generation and apoptosis resistance in the synovium of rheumatoid arthritis.^297^ Histone deacetylases (HDAC) are another Star family that has been widely studied. Studies have proven that HDAC1 participates in producing pro-inflammatory factors, and the elimination of HDAC1 in T cells has a protective function on mice with collagen arthritis.^298^ HDAC inhibitors can inhibit the activation of FLS, and the HDAC1 and HDAC2 expression in RA synovial fibroblasts (RA-SF) is higher than that in OA synovial fibroblasts (OASF).^299^

Research from our group has demonstrated that HDAC6 protein levels in the adjuvant-induced arthritic rats’ synovium tissues are increased.^300^ Interestingly, in animal RA models, HDAC inhibitors can improve joint swelling and synovial inflammation and reduce RA symptoms. This evidence provides novel ideas for RA treatment.^301,302^ Our team also found that in PDGF-induced FLS, the expression of the Jumonji C histone demethylase family (JMJD3) is increased through the Akt signaling pathway, meanwhile, the migration and proliferation ability of FLS is weakened after inhibiting or silencing of JMJD3. Cumulatively, this reduced the rates of CIA.^303^

Additionally, Non-histone modifications were also observed; for example, Yin et al.^304^ found that expression of Jmjd1c (a member of the JmjC domain histone demethylase) in B cells was found to protect mice from rheumatoid arthritis. In human B cells with RA, the expression levels of Jmjd1c are inversely correlated with plasma cell levels and disease severity, and Jmjd1c demethylates STAT3 but not histones to inhibit plasma cell differentiation. Meanwhile, our group found histone methyltransferase Smyd2-mediated TRAF2 methylation promotes inflammatory diseases (including RA) through the NF-κB signaling pathway.^305^ which might also provide some insight for treatment strategies.

### DNA methylation and RA

DNA methylation is under DNA methyltransferase (DNMT) catalytic action and transfers the methyl group from S-adenosine methionine (SAM) to the DNA sequence. DNA methylation occurs at the Cytosine of CpG (Cytosine-phosphoric acid guanine) islands to produce 5MC, most of which are located in the promoter region.^306^ Abnormal hypermethylation of the CpG islands will prevent transcription factors from binding to the promoter and lead to gene silencing.^307,308^ DNA methylation is considered a potential therapeutic target due to its reversibility. Indeed, DNA methylation levels in synovial tissue of RA and OA patients are not significantly different. However, DNA methylation levels are reported to be lower in RA patients with peripheral blood mononuclear cells (PBMC).^309^ Furthermore, in PBMC, abnormal cytosine methylation occurs in the promoter regions of IL-6 and IL-10, affecting transcription.^310,311^ Indeed, studies have shown that DNA methylation levels in T cells and monocytes in RA patients are lower than those in healthy subjects.^312,313^ Hypermethylation of the promoter region act as a marker of heterochromatin, which affects the binding of DNA to transcription factors and inhibits gene transcription. The specific recombination of methyl groups in the synovial fibroblasts of RA occurs during the development of the disease. DNA methylation reduction is often found in highly proliferative tissues and is related to the methyl group donor molecule, SAM. In addition, the hypomethylation of DNA leads to the increased expression of extracellular matrix proteins, growth factors/receptors, matrix-degrading enzymes, and adhesion molecules. Therefore, in proliferating tissues, these are usually used as markers to identify whether cell proliferation is occurring. The methylation level of cells seems to be affected by the inflammatory environment in which they are located. Some studies stimulated FLS with IL-1 and TNF-α, and the results show that the methylation level of RA-FLS is significantly lower than that of OA-FLS. Moreover, 5-methylcytosine levels were increased.^314,315^ In RA-FLS, the T-box transcription factor 5 promoter region differs from OA-FLS in methylation status.

Furthermore, the promoter region of its downstream gene Chemokine CxCL12 also shows high rates of hypomethylation in RA-FLS.^315^ Cribbs et al. revealed that the compromised function of Treg in RA patients is associated with the hypermethylation of a specific region in the promoter of the cytotoxic T-lymphocyte-associated protein 4 (CTLA-4;−658 CpG) as compared with healthy controls.^316^ DNA hypermethylation prevents the binding of NFATc2 to CTLA-4 and decreases CTLA-4 expression. Consequently, Treg cells lose their function to promote the activation and expression of tryptophan-degrading enzyme indoleamine 2,3-dioxygenase (IDO). In turn, these cells fail to activate the immunomodulatory pathway. These results indicate that small changes in methylation can impact different cell types in RA.

### MicroRNAs and RA

MicroRNAs occupy an important position in modifying non-coding RNAs; MicroRNA (miRNA) is a kind of non-coding RNA molecule.^317,318^ Initially, the miRNA gene is transcribed to form a primary miRNA in the nucleus, cleaved by Drosha to form a precursor -miRNA. By Exportin-5, miRNA is exported to the cytoplasm and cleaved by Dicer to form mature miRNA duplexes, which are then unfolded, and a miRNA strand is added to the RNA-induced silencing complex.^319,320^ Combining miRNAs and their target mRNAs result in transcriptional repression or mRNA decay.^321^ MicroRNAs have been implicated in the occurrence and progression of many diseases, especially cancer,^322^ but now research points focus on their roles in immunological diseases.^323^ The expression of various miRNAs changed during the development of RA, including miR-146a, miR-155, miR-222, miR-223, miR-203,^324–329^ and miR-132, miR-155, and miR-146a might be potential biomarkers of response to methotrexate treatment in patients with RA.^330^ Some miRNAs can influence RA by regulating the function of FLS. For instance, miR-203 is upregulated in RA-FLS and induces RA by promoting the generation of MMP-1 and IL-6.^331^ The expression of miR-19 was upregulated in TNF-α-stimulated FLS, and the inflammatory response was mediated by regulating TLR2, IL-6, and MMP-3.^332,333^ Many miRNAs are also down-regulated in RA, for example, miR-10a by RAFLS, and can regulate the production of inflammatory factors through the NF-κB signaling pathway.^334^ The expression of miR-19 decreased in lipopolysaccharide-stimulated RAFLS, and its anti-inflammatory effect is induced by the regulation of IL-1β, IL-6, and other inflammatory factors.^335^ In animal models, miR-124a can inhibit RA symptoms in rat AIA models by reducing synovial cell proliferation and alleviating cartilage or bone destruction.^336^ MicroRNAs levels before and after anti-TNF-α combination therapy are potential new biomarkers for monitoring and predicting intervention outcomes. For example, miRNA-23-3p, miRNA-16-5p, miRNA125b-5p, miRNA-146A-5P, miRNA-126-3p, and miRNA-223-3p were found to be significantly upregulated after anti-TNF-α treatment. Interestingly, only responders showed an increase in these miRNAs after treatment, consistent with a decrease in C-reactive protein (CRP), rheumatoid factor (RF), TNF-α, interleukin (IL)-6, and IL-17.^337^

## Targeted therapy for RA

Treatment for rheumatoid arthritis could help relieve pain, reduce joint inflammation, prevent or slow joint injury, reduce disability and keep patients as active as possible. Although rheumatoid arthritis has no cure, an early drug intervention could reduce the risk and pain of joint damage and slows the progression of the disease. In general, non-steroidal anti-inflammatory drugs (NSAID), glucocorticoids (GCs), and disease-modifying anti-rheumatic drugs (DMARDs) are applied in clinical RA treatment, and some cutting-edge technology therapies are emerging for targeted therapy.

DMARDs are subdivided into conventional synthetic DMARDs (csDMARDs), biologic DMARDs (bDMARDs), and targeted synthetic DMARDs (tsDMARDs).^338^ The principal approved drugs, the drugs currently being assessed in clinical trials, and various pre-clinical drugs for RA treatment are highlighted in Table 1.

**Table 1:** The main approved drugs, potential clinical trial drugs, and pre-clinical drugs for RA treatment

Types	Name	Targets/mechanisms	Indications	Status	References
NSAIDs	Naprelan (naproxen sodium)	Suppressing COX activity, thereby inhibiting PGs synthesis, and exerting antipyretic and analgesic effects	Treating mild to moderate pain, swelling, and joint stiffness caused by rheumatoid arthritis	Approved by FDA	^345^
	Mobic (meloxicam)	Selectively inhibiting COX2 and weakly inhibiting COX-1	Relieving pain, swelling, and stiffness of the joints caused by rheumatoid arthritis	Approved by FDA	^339,341,342^
	Duexis (ibuprofen and famotidine)	Ibuprofen: COX-1 and COX2 inhibitor Famotidine: histamine blocker	Ibuprofen: treating the symptoms and signs of rheumatoid arthritis Famotidine: reducing the risk of upper gastrointestinal ulcers caused by chronic use of ibuprofen	Approved by FDA	^344^
GCs	Rayos (prednisone) delayed-release tablets	Lowering the activity of the immune system through the active modified-release formulation of prednisone	Cutting down on pain, redness, and swelling in the body	Approved by FDA	^347–350^
csDMARDs	Rasuvo (methotrexate)	Antifolate and immunosuppressant, inhibition of AICAR transformylase	Management of adults with severe and active rheumatoid arthritis	Approved by FDA	^352–354^
	Arava (leflunomide)	Suppressing the activity of dihydroorotate dehydrogenase (DHODH), thereby inhibiting pyrimidine synthesis in activated lymphocytes	Treating adult moderate to severe rheumatoid arthritis and psoriatic arthritis	Approved by FDA	^356,357^
	Azulfidine (sulfasalazine)	Inhibiting folate metabolizing enzymes	RA Patients who have inadequate responses to salicylates or other NSAIDs	Approved by FDA	^358–361^
bDMARDs	Kineret (anakinra)	IL-1Ra	Modest efficacious therapy for moderate to severe active rheumatoid arthritis (RA)	Approved by FDA	^369–372^
	Actemra (tocilizumab)	IL-6 Ra	Reduction of signs and symptoms of moderately to severely active rheumatoid arthritis	Approved by FDA	^362,364–366^
	Kevzara (sarilumab)	IL-6 Ra	Moderately to severely active and progressive rheumatoid arthritis in adults	Approved by FDA	^367,368^
	Enbrel (etanercept)	Binding to TNF and blocking its interaction with TNF receptors on the cell surface	Reducing pain and joint damage in patients with moderate to severe rheumatoid arthritis.	Approved by FDA	^373–375^
	Humira (adalimumab)	TNF-α antagonist/ TNF inhibitor	To ameliorate the signs and symptoms of moderate to severe rheumatoid arthritis (RA) in adults	Approved by FDA	^376–378^
	Remicade (infliximab)	Blocking the action of TNF-α	Preventing the joint damage progression, and improving physical function in adult patients suffering from moderately to severely active rheumatoid arthritis	Approved by FDA	^379,380,487^
	Simponi (golimumab)	TNF-α inhibitor	Therapy for adults with moderate to severe rheumatoid arthritis	Approved by FDA	^383,384^
	Orencia (abatacept)	Blocking the activity of T cells by binding to CD86 and CD80, thus blocking the interaction with CD28.	Relieving the pain, swelling, and joint injury caused by rheumatoid arthritis	Approved by FDA	^385–388^
	Rituxan (rituximab)	Anti-CD20 monoclonal antibody: Targeting a transmembrane protein, CD20, present on the pre-B and mature B-lymphocytes surface	Used with MTX to treat moderately to severely active rheumatoid arthritis	Approved by FDA	^389–393^
tsDMARDs	Iguratimod (IGU)	NF-kB inhibitor/COX2 inhibitor, decreasing the immunoglobulins and cytokines production, thereby mediating T lymphocytes subsets	Function on synovial tissue of rheumatoid arthritis	Approved in China and Japan	^415–419^
	Rinvoq (upadacitinib)	JAK1 selective inhibitor	Treatment of moderate to severe rheumatoid arthritis, including the patients who presented an inadequate response or intolerance to methotrexate	Approved by FDA	^378,385,404–406^
	Olumiant (baricitinib)	JAK1/JAK2 inhibitor	Relieving the symptoms of moderate to severe rheumatoid arthritis, including the patients who did not respond well to one or more TNF inhibitor treatment	Approved by FDA	^400–403^
	Xeljanz (tofacitinib)	JAK1/JAK3 inhibitor	Treatment for moderately to severely active rheumatoid arthritis, and is most commonly used after taking methotrexate and TNF inhibitors.	Approved by FDA	^395–399^
	Jyseleca (Filgotinib)	JAK1 selective inhibitor	Therapy for moderate to severe active rheumatoid arthritis (RA) in adults, including those who did not respond or tolerate it well to DMARDs	Approved in EU and Japan	^407–410^
	Smyraf (Peficitinib)	Janus JAK1/2/3 inhibitor, suppressing the activation of cytokine signaling pathways	Therapy for rheumatoid arthritis in patients who respond inadequately to conventional treatment	Approved in Japan	^411–414^
	VX-509 (Decernotinib)	JAK3 selective inhibitor	Improving the signs and symptoms of rheumatoid arthritis can be used in combination with methotrexate	Phase 2/3 clinical trial (NCT01830985)	^427–429^
	Jakafi® (Ruxolitinib)	JAK1/2 inhibitor	Rheumatoid arthritis	Phase 2 clinical trial (NCT00550043)	^424^
	SHR0302	Highly selective JAK1 inhibitor	Rheumatoid arthritis	Phase 3 clinical trial (NCT04333771)	^426^
	Fenebrutinib	selective BTK Inhibitor	Efficacy was shown in patients with rheumatoid arthritis who had an inadequate response to MTX	Phase 2 clinical trial (-)	^237,423^
	VX-702	P38 MAPK inhibitor	Rheumatoid arthritis, but it may show no sustained suppression in disease progression	Phase 2 clinical trial (NCT00205478)	^430^
	SCIO-469	P38 MAPK inhibitor	Rheumatoid arthritis, but it exhibited no better efficacy compared with a placebo in patients	Phase 2 clinical trial (NCT00043732)	^431^
	PH-797804	P38 MAPK inhibitor	Rheumatoid arthritis	Phase 2 clinical trial (NCT00620685)	^432^
	SB-681323	P38 MAPK inhibitor	Rheumatoid arthritis	Phase 2 clinical trial (NCT00320450)	^433^
	BMS-582949	P38 MAPK inhibitor	Rheumatoid arthritis	Phase 2 clinical trial (NCT00605735)	^434^
	GS9901	Selective PI3Kδ inhibitor	Rheumatoid arthritis	Pre-clinical study	^435^
	PBT-6	PI3KC2γ inhibitor	Rheumatoid arthritis	Pre-clinical study	^436^
	ZSTK474	PI3K inhibitor	Rheumatoid arthritis, it may reduce synovial inflammation and bone destruction in patients	Pre-clinical study	^437^
	Rapamycin	mTOR inhibitor	Rheumatoid arthritis	Pre-clinical study	^438,439^
	LY411575	Notch-1/3 inhibitor	Rheumatoid arthritis	Pre-clinical study	^440^
	STA-21	STAT3 inhibitor	Rheumatoid arthritis	Pre-clinical study	^441^
Epi-drugs	Azacitidine	DNMT inhibitor, diminishing the production of inflammatory cytokines (IL-6 and TNF-α) in RAFLS	Rheumatoid arthritis	Pre-clinical study	^442^
	Decitabine	DNMT inhibitor, reducing the release of Th1 and Th17 pro-inflammatory cytokines in the CIA mouse model	Rheumatoid arthritis	Pre-clinical study	^443^
	Zebularine	DNMT inhibitor, sustainably diminishing the severity of arthritis and promoting the generation of Treg	Rheumatoid arthritis	Pre-clinical study	^444,447^
	Epigallocatechin-3-gallate (EGCG)	DNMT inhibitor, decreasing the production of IL-6, IL-8, and MMP-2 and selectively suppressing COX2 expression in human RAFLS	Rheumatoid arthritis	Pre-clinical study	^445^
	MS-275 / SAHA	HDAC1, HDAC3, Class I, and Class II HDAC inhibitor, restraining LPS-induced nuclear aggregation of NF-κB p65 in synovial fibroblastic E11 cells and THP-1 monocytes	Rheumatoid arthritis	Pre-clinical study	^451^
	MI192	HDAC3 inhibitor, decreasing LPS-induced IL-6 production in PBMCs isolated from RA patients	Rheumatoid arthritis	Pre-clinical study	^448^
	Trichostatin A (TSA)	Class I and Class II HDAC, lessening IL-6 mRNA stability in RAFLS	Rheumatoid arthritis	Pre-clinical study	^449^
	Nicotinamide	SIRT1-7 inhibitor, reducing LPS-induced IL-6 and TNF-α expression in macrophages; triggering apoptosis in macrophages	Rheumatoid arthritis	Pre-clinical study	^450^
	Largazole	Class I HDAC inhibitor, enhancing TNF-α-induced VCAM-1 and ICAM-1 expression in RASF; blocking TNF-α-induced MMP-2 activity; regulating Class II HDAC expression levels	Rheumatoid arthritis	Pre-clinical study	^452^
	MPT0G009	Class I HDAC and HDAC6 inhibitor, reducing PGE2 and IL-6 production in RAFLS; refraining osteoclast formation; diminishing paw swelling and arthritis scores in AIA rats	Rheumatoid arthritis	Pre-clinical study	^453^
	NK-HDAC1	HDAC1inhibitor, decreasing proliferation rates of RAFLS and suppressing TNF-α-induced IL-6 and MMP-3 release; promoting apoptosis of synoviocytes and cutting down on disease progression in CIA mice	Rheumatoid arthritis	Pre-clinical study	^454^
	CKD-L, tubA	HDAC6 inhibitor, blocking the activity of TNF and IL-1β, and increasing IL-10 level in PBMCs from RA patients; decreasing TNF production in THP-1 cells; reducing the arthritis score in CIA mice	Rheumatoid arthritis	Pre-clinical study	^455^
	CKD-506	HDAC6 inhibitor, lessening TNF-α and IL-6 production by activated PBMCs from RA patients; decreasing MMP-1, MMP-3, IL-6, and IL-8 secretion by activated FLS; moderating the severity of arthritis in a murine model of AIA	Rheumatoid arthritis	Pre-clinical study	^456^
Other	SPRC	Ameliorating inflammatory response via Nrf2-ARE signaling pathway and HDAC6/MyD88/NF-κB signaling pathway in RA model	Rheumatoid arthritis	Pre-clinical study	^300,462,464,465^

### Approved drugs

In clinical, NSAIDs like Naprelan (naproxen sodium), Mobic (meloxicam), and Duexis (ibuprofen and famotidine) inhibit cyclooxygenase (COX) activity, thereby inhibiting prostaglandins (PGs) synthesis and producing antipyretic and analgesic effects used for relief of the symptoms and pain of rheumatoid arthritis.^339–345^ In the case of excessive use or overtime use of NSAIDs, there will be leukopenia, thrombocytopenia, etc., and digestive tract lesions, such as stomach pain, gastric ulcer, and even ulcer bleeding (so famotidine in Duexis is used for inhibition of gastric secretion), liver damage, kidney damage, etc.^346^ GCs and Rayos (prednisone) delayed-release tablets also help relieve symptoms and benefit RA patients.^347–350^ However, the treatment of hormone drugs can easily lead to side effects like endocrine disorders, osteoporosis, obesity, and decreased immunity.^351^

Methotrexate (MTX) is the most commonly used csDMARDs and has been regarded as a first-line drug for years. The chemical structure of MTX is similar to folic acid, an antifolate drug. This molecule was originally used for tumor chemotherapy, and low dosages are used for rheumatoid therapy. MTX appears to involve multiple mechanisms, including inhibition of interleukin-1-β binding to the cell-surface receptors,^352,353^ the inhibition of purine metabolism enzymes, leading to adenosine accumulation and inhibition of T-cell activation and the expression of adhesion molecule. Other studies indicate increased sensitivity of activated T cells to CD95, the selective downregulation of B cells, and the inhibition of methyltransferase activity contributing to the inactivation of enzymes related to immune system function.^354,355^ Leflunomide is a prodrug, which can be rapidly converted into active metabolites in the body after taking it to inhibit the dihydroorotate dehydrogenase activity and affect the synthesis of pyrimidine in activated lymphocytes, thereby exerting an anti-inflammatory effect.^356,357^ Azulfidine (sulfasalazine) has the dual effects of anti-inflammatory and antibacterial and could also inhibit the synthesis of immune complexes and rheumatoid factors, thereby alleviating the immunopathological damage of rheumatoid arthritis.^358–361^

The bDMARDs, Actemra (tocilizumab) and Kevzara (sarilumab) are interleukin-6 (IL-6) receptor antagonists,^362–368^ and the IL-1 receptor antagonist Kineret (anakinra)^369–372^ were approved by the FDA for moderate to severely active rheumatoid arthritis adult patients. Enbrel (etanercept) relieves inflammation in RA patients by binding tumor necrosis factor (TNF).^373–375^ Humira (adalimumab) is also working as a tumor necrosis factor (TNF) blocker,^376–378^ and Remicade (infliximab) blocks the activity of TNF-α.^379–381^ In addition, Simponi (golimumab) binds to both the transmembrane and soluble bioactive forms of human TNF-α, thus preventing TNF-α binding to its receptors^382–384^ in the case of treating RA. Orencia (Abatacept) is a selective costimulatory regulator that inhibits T-cell (T lymphocyte) activation by binding to CD86 and CD80, thus blocking the interaction with CD28.^385–387^ This interaction provides the necessary costimulatory signals to fully activate T lymphocytes, which are participated in the pathogenesis of RA.^388^ Rituxan (Rituximab) is a monoclonal antibody targeting the surface CD20 antigen of pre-B and mature B-lymphocytes. Upon binding to CD20, rituximab leads to antibody-dependent cell-mediated cytotoxic (ADCC) and complement-dependent cytotoxic (CDC) lysis of B cells.^389–392^ B cells are proven to play a role in the pathogenesis of RA and related chronic synovitis, including the production of RF or other autoantibodies, antigen-presenting, T-cell activation, and related pro-inflammatory cytokine generation.^393^

The tsDMARDs are the latest drugs for RA treatments; The US Food and Drug Administration (FDA) approved some JAK inhibitors in clinical use. These small molecules help prevent an individual’s immune system from producing certain enzymes that stimulate inflammation.^394^ Tofacitinib, a JAK pathway inhibitor developed in 2012, is FDA-approved and marketed under the brand name Xeljanz. Tofacitinib citrate is approved for medical use “to treat adults with moderately to severely active rheumatoid arthritis who have had an inadequate response to or are intolerant of methotrexate”.^395–399^ Baricitinib, sold under Oluminant, acts as a JAK1 and JAK2 inhibitor. In May 2018, the FDA-approved baritinib for treating moderate-to-severe active rheumatoid arthritis patients who have not responded adequately to treatment with one or more TNF antagonists.^400–403^ Upadacitinib, marketed under the brand name Rinvoq, is a JAK inhibitor drug FDA-directed for the treatment of adults with psoriatic arthritis and moderate to severely active rheumatoid arthritis who do not or may not respond to methotrexate.^404–406^ JAK1 inhibitor Filgotinib has been approved for the treatment of RA in the European Union and Japan.^407–410^ Peficitinib is a JAK3 inhibitor for treating RA recently approved in Japan.^411,412^ Peficitinib attenuates RA symptoms and inhibits joint destruction in Japanese RA patients who do not respond adequately to MTX.^413,414^ Iguratimod, which inhibits the activation of NF-κB, is a novel DMARDs approved for the treatment of RA in Japan and China.^415–419^

NSAIDs are generally only used in the early stage to reduce symptoms of the disease, or until the diagnosis of RA is established, methotrexate is usually combined with GCS for a period of time to control inflammation and gradually reduces the use of GCs, under the initial treatment regimen, about 30–50% of patients with rheumatoid arthritis are in remission, and other csDMARDs are usually added if treatment purpose is not achieved within 3–6 months with methotrexate monotherapy.^26,420^ If treatment has not achieved the desired results, it is usually combined with methotrexate and biological or targeted synthetic DMARDs.^421^ This combination therapy could control an additional 30%- 40% of patients with rheumatoid arthritis.^5^

Common side effects of currently available DMARDs, in addition to cytopenia, liver damage, and elevated cholesterol, both targeted synthesis and biological DMARDs lead to increased frequency of infection, which may be caused by inhibition of their respective inflammatory mediators.^338^

Of note, targeted synthetic or biologics DMARDs are not supposed to be regarded as first-line therapy because most patients who respond to these drugs also respond to methotrexate treatment alone. Meanwhile, methotrexate is associated with lower cost, lower side effects, and infection frequency compared with targeted synthetic or biological DMARDs.^422^ Individual patient disease status and treatment outcomes are continually reassessed throughout treatment; it is essential to make adjustments in time.

### Drugs in clinical trials

Cohen et al. are developing an experimental drug called Fenebrutinib that blocks the action of Bruton’s tyrosine kinase (BTK). When recently assessed in a phase 2 trial, Fenebrutinib effectively treated patients with RA who had no response to other therapies. Compared with the popular RA drug Humira (Adalimumab), Finebrutinib showed similar efficacy.^423^ Although more research is needed, scientists are excited about the potential of BTK inhibitors to help RA patients. Similarly, JAK inhibitors are still popular targets for developing RA drugs, and several other JAK inhibitors have proceeded into clinical trials for RA treatment. Ruxolitinib is a selective JAK1/JAK2 inhibitor^424^ and has been demonstrated for treating psoriasis and myeloproliferative diseases.^425^ Rusolitinib is generally safe in patients of RA and normal volunteers and has now completed phase 2 clinical trials (NCT00550043). A recent study has assessed the safety and efficacy of a selective inhibitor of JAK1 SHR0302^426^ in rheumatoid arthritis patients, and this molecule has now entered a Phase 3 clinical trial (NCT04333771). In addition, a phase 2/3 study is underway to assess the safety and long-term efficacy of the JAK3 inhibitor VX-509^427–429^ in rheumatoid arthritis patients (NCT01830985). These drugs are expected to be available soon.

In recent years, many P38 kinase inhibitors have entered clinical trials, unfortunately, no effective inhibitors have been identified. The P38 MAPK inhibitor, VX-702, has shown mediocrity clinical efficacy and transiently inhibits inflammatory factors. However, but appears not to promote sustained inhibition of the chronic inflammation in RA.^430^ Other compounds like SCIO-469 show no differences in efficacy when compared to the placebo treatment in RA patients.^431^ Moreover, the therapeutics, Ph-797804,^432^ SB-681323,^433^ and BMS-582949,^434^ inhibitors of P38, are now in RA treatment clinical trials, but results from these interventions have yet to be reported.

### Drugs in pre-clinical studies

PI3K takes part in inflammatory processes and is a potential therapeutic target for RA. GS9901 is a selective oral PI3Kδ inhibitor that proved efficacious in a RA animal model.^435^ The inhibition of PI3KC2γ expression in macrophages and synovial fibroblasts by PBT-6 suggests that it can be used as a novel inhibitor of PI3KC2γ in inflammatory diseases, including rheumatoid arthritis.^436^ Inhibition of PI3K by ZSTK474 may inhibit bone destruction and synovial inflammation in RA patients, and the Inhibition efficiency of ZSTK474 is much better than that of LY294002, a commonly used PI3K inhibitor.^437^ The mTOR inhibitor rapamycin may apply in the treatment of RA, aiming to reduce FLS-mediated joint injury and erosive changes. The combination of mTOR inhibitor and vitamin D3 prevents bone destruction in RA.^438,439^ Notch signaling inhibitor LY411575 inhibits −1 and Notch-3 for treating collagen-induced arthritis (CIA) in rats.^440^ Ahmad et al^441^ have reported that STAT3 inhibitor STA-21 reduced the expression of TNF-α and IL-6 in the peripheral blood of collagen-induced arthritis rats and increased the expression of IL-27 produced by CD14+ cells.

Many researchers have indicated that DNA methylation inhibitors like Azacitidine (5’-AzaC) have the potential to inhibit RA progression. In Table 1, we list several DNMT inhibitors that have been investigated in RA drugs (trials or studies). Azacitidine blocked the release of inflammatory cytokines (TNF-α and IL-6) in RAFLS.^442^ Decitabine Exhibited a decreasing production of Th1 and Th17 pro-inflammatory cytokines and can reduce anti-type II collagen autoantibodies. In the CIA mouse model,^443^ Zebularine produced a sustained reduction in the severity of arthritis and promoted the generation of Treg.^444^ Epigallocatechin-3 gallate (EGCG) inhibits MMP-2, IL-6, and IL-8 production and selectively inhibits COX2 expression in human RAFLS.^445^ Importantly, these drugs have been shown to inhibit the activation of DNMTs in RA-related studies. 5’-AzaC, zebularine, and decitabine are the same class of drugs that belong to nucleoside-derived inhibitors, which were initially investigated and approved for the treatment of cancer.^446,447^ With the development of nucleoside-derived inhibitors, they were also studied for use in treating RA patients. It was confirmed that treatment with 5’-AzaC decreased the expression of inflammatory cytokines (i.e., TNF-α and IL-6) in RAFLS.^442^ Furthermore, 5’-AzaC elevated the anti-inflammatory cytokine IL-10 expression in PBMCs isolated from RA patients. This finding was related to the hypomethylation of the IL-10 promoter.^311^ In a murine CIA model, decitabine showed inhibitory effects towards the anti-type II collagen autoantibodies production and Th1 or Th17 pro-inflammatory cytokines.^443^

Different HDAC inhibitors target a few members of the HDAC enzyme family and were claimed to have beneficial effects in the treatment of RA. The selective HDAC3 inhibitor MI192 was proven to inhibit the expression of TNF-α and IL-1β induced by LPS in PBMCs, which were derived from healthy donors and RA patients.^448^ TSA, a Class I and Class II HDAC inhibitor, induced a remarkable decrease in nuclear retention of NF-κB in RAFLS in the presence of IL-1β stimulation, resulting in the temporal reduction of IL-6 mRNA accumulation.^449^ In another report, nicotinamide, a Class III HDAC inhibitor reduced LPS-stimulated IL6 and TNF-α expression and TNF-α-induced expression of IL-6 in macrophages isolated from RA patients.^450^ Furthermore, MS-275 and SAHA, then on-specific HDAC inhibitors suppressed the NF-κB p65 nuclear accumulation, induced by LPS in human RA synovial fibroblastic E11 cells THP-1 monocytes, leading to a reduction in pro-inflammatory cytokines.^451^ Largazole, a Class I HDAC inhibitor, Enhanced TNF-α-induced expression of VCAM-1 and ICAM-1 in RASF; inhibited TNF-α-induced MMP-2 activity; modulated Class II HDAC expression levels.^452^ MPT0G009, a Class I HDAC and HDAC6 inhibitor, Reduced PGE2 and IL-6 secretion in RAFLS; reduced paw swelling; reduced osteoclast formation and arthritis scores in AIA rats.^453^ NK-HDAC1, HDAC1inhibitor, reduced proliferation rates of RAFLS and suppressed TNF-α-induced MMP-3 and IL-6 secretion; increased apoptosis of synoviocytes and delayed the progression of disease in CIA mice.^454^ Recently, HDAC6-specific inhibitors CKD-L and tubA inhibited the expression of IL-1β and TNF-α and increased the IL-10 expression in PBMCs from RA patients.^455^ Meanwhile, these compounds inhibited TNF-α secretion in THP-1 cells and reduced the arthritis score in CIA mice. CKD-506, an HDAC6 inhibitor, Reduced the production of IL-6 and TNF-α by activated PBMCs from RA patients; inhibited the production of IL-8, IL-6, MMP-1, and MMP-3 by activated FLS; inhibited the severity of arthritis in a murine model of AIA.^456^ Interestingly, these studies could not show an association between the use of these HDAC inhibitors and the deacetylation of H3 and H4. Since p53 has been reported as a non-histone protein that can be acetylated by HAT,^457^ a drive in research to study other non-histone targets of HDACs and HATs, such as c-MYC, NF-κB, STAT3, α-tubulin has occurred.^458^ The non-histone proteins acetylation and deacetylation play roles in all sorts of human diseases, including RA, cancer, and Parkinson’s disease (PD).^459–461^ Therefore, targeting non-histone proteins might be a promising therapeutic strategy for the treatment of RA.

Additionally, Our research has revealed that the hydrogen sulfide (H_2_S) donor S-propargyl-cysteine (SPRC, named also as ZYZ-802) could alleviate inflammatory response and inhibit HDAC6 expression in vivo via the HDAC6/MyD88/NF-κB signaling pathway,^300^ or Nrf2-ARE signaling pathway.^462^ At the same time, we also found that CSE/H_2_S can reduce the expression of JMJD3 by inhibiting transcription factor SP-1 and alleviating arthritis.^463^ SPRC might serve as a potential drug for RA treatment. We have developed two sustained-release donors of hydrogen sulfide,^464,465^ which has solved the problem of hydrogen sulfide release too fast in conventional formulations. ZYZ-802 is filing for clinical trials by CFDA and FDA now.

### Cutting-edge technology therapy

Some cutting-edge technologies are emerging for RA treatment, for example: Targeting protein degradation as a new therapeutic approach by using Proteolysis-targeting chimeras (PROTAC) technology to address diseases caused by abnormal expression of pathogenic proteins. PROTAC molecule can bind both the E3-ubiquitin ligase and the target protein, thereby causing the target protein ubiquitination and degradation.^466,467^ However, PROTAC delivery and bioavailability remain the biggest obstacles to clinic.^468^ Addressing these issues will be the focus of many laboratories in the coming years. PROTAC -mediated degradation of JAK has been proposed as a novel and promising therapeutic strategy for rheumatoid arthritis.^469^

Nanoparticles are new and promising drug delivery systems (DDSs) that are designed to deliver a specific dose of the desired medicine to a particular part of the body. They make it safe to increase the bioavailability of drug compounds by allowing drug-controlled release over time. Targeted drug delivery nanomaterials for RA therapy focus on efficacy at the lesion site through local delivery of active ingredients while sparing normal cells and tissues from off-target toxicity. Polylactic-co-glycolic acid (PLGA) is the most widely used nanoparticle because of its biocompatibility, and the FDA has approved PLGA as a drug carrier,^470^ some pre-clinical studies indicated a combination treatment of MTX-loaded PLGA nanoparticles and near-infrared irradiated showed a durable and superior therapeutic effect in suppressing arthritis compared with MTX single administration.^471^ Yang et al. announce the first example of RA treatment using bioactive nanoparticles without any drug loading and highlight the role of folic acid-modified silver nanoparticles (FA-AgNPs) through M1 macrophage apoptosis and M1-to-M2 Macrophage repolarization for targeted RA therapy.^472^

A research team from St. Louis, USA, used CRISPR-Cas9 genome editing technology to transform induced pluripotent stem cells (iPSCs) and constructed cartilage stem cells called “SMART” (Stem cells Modified for Autonomous Regenerative Therapy) in which cells are implanted with a synthetic gene circuit that is regulated by IL-1 to produce an IL-1 receptor antagonist (IL-1Ra). IL-1 promotes inflammation in arthritis by activating inflammatory cells in the joints. When inflammation occurs, intracellular gene circuits that sense changes in endogenous IL-1 cytokine levels are activated to secrete therapeutic levels of IL-1Ra.^473^ If one therapeutic drug works better than another in a particular patient, it may be possible to develop individualized therapies by reprogramming chondrocytes.

Although RA remains incurable, the development of DMARDs and refined treatments make RA a generally manageable disease. By using different combinations of DMARDs, many patients experience remission of symptoms. However, there are still a large number of patients still do not respond to available therapies to date, indicating the necessity to develop new drugs and treatment strategies. It is hoped that in the near future, some pre-clinical drugs and strategies will successfully move toward clinical studies, providing more options for RA patients.

## Conclusions and prospects

Rheumatoid arthritis is a systemic chronic autoimmune disease,^474^ characterized by symmetrical articular synovitis. The repeated attacks of articular synovitis and the formation of synovial pannus cause the erosion and destruction of the cartilage and subchondral bone in the affected joints, eventually leading to various deformities of the affected joints and the dysfunction of joint function.^475–478^ Generally, NSAIDs, GCs, and DMARDs are used for clinical RA treatment. However, they can only delay the disease progression or improve inflammatory symptoms. Furthermore, since the treatment of RA is a long-term process, the side effects of these drugs are inevitable, including immunosuppression, gastrointestinal ulcers, osteoporosis, nausea, fatigue, cytopenia, rashes, liver damage, infections, and psoriasis.^479–482^ Therefore, it is an urgent need to develop novel therapeutic strategies that enhance efficacy and reduce toxicity.

During the disease progression of RA, some pro-inflammatory cytokines trigger signal transductions associated with RA, which lead to the recruitment of innate and adaptive immune cells and the activation of synovial cells. These systems release inflammatory mediators, including IL-1, IL-6, and TNF-α, leading to synovial inflammation and exacerbating disease progression.^483^ A deeper understanding of the involvement of abnormal signal transduction in RA will provide us with novel strategies to prevent and treat this disease class. With the approval of JAK inhibitors for the treatment of RA,^484^ kinase inhibitors have become a hot spot in drug research. In this review, we summarized the signaling pathways involved in the RA pathogenesis, new potential targets, and associated inhibitors, such as MAPK, WNT, PI3K/AKT, SYK, and JAK/STAT pathways, respectively. We also indicate new targets such as NAV2. Furthermore, it is raised that P38 inhibitors applied in the treatment of RA are not ideal.^430,431^ These highlights future challenges in treating RA and the need to identify new specific targets to drive developments in the synthesis of newer selective inhibitors.

Several cutting-edge technologies are appearing for RA therapy, for instance: Targeting protein degradation as a new therapeutic approach by using Proteolysis-targeting chimeras (PROTAC) technology; Nanoparticles were used for drug targeting and sustained-release delivery; CRISPR-Cas9 genome editing technology et.al. Advances in RA treatment have taught us that “one size does not fit all” and that personalized therapy is now the consensus goal.^473,485,486^

In closing, the current review highlights specific signal transduction pathways and molecular targets that may hold promise in the treatment of RA, also raised the developments in new drugs for use and prospect some cutting-edge technologies in treating RA, hope to provide new ideas for RA’s therapy in the future.

## Methods

We reviewed the literature on rheumatoid arthritis up to 2022. PubMed was searched using the terms “rheumatoid arthritis” plus “signaling pathways”, “molecular mechanisms”, “genetic factors”, “epigenetics”, and “therapeutic interventions”. A literature review of the retrieved papers was presented herein.
